# Transcriptome analyses reveals the dynamic nature of oil accumulation during seed development of *Plukenetia volubilis* L.

**DOI:** 10.1038/s41598-020-77177-w

**Published:** 2020-11-24

**Authors:** Guo Liu, Zhihua Wu, Yan Peng, Xiuhua Shang, Yaojian Xie, Roger J. Arnold

**Affiliations:** China Eucalypt Research Centre (CERC), Zhanjiang, 524022 Guangdong China

**Keywords:** Gene expression, Gene regulation, Cell biology, Molecular biology, Plant sciences

## Abstract

Sacha inchi (*Plukenetia volubilis* L.) is a shrub native to Amazon rainforests that’s of commercial interest as its seeds contain 35–60% edible oil (dry weight). This oil is one of the healthiest vegetable oils due to its high polyunsaturated fatty acid content and favourable ratio of omega-6 to omega-3 fatty acids. De novo transcriptome assembly and comparative analyses were performed on sacha inchi seeds from five stages of seed development in order to identifying genes associated with oil accumulation and fatty acid production. Of 30,189 unigenes that could be annotated in public databases, 20,446 were differentially expressed unigenes. A total of 14 KEGG pathways related to lipid metabolism were found, and 86 unigenes encoding enzymes involved in α-linolenic acid (ALA) biosynthesis were obtained including five unigenes encoding FATA (Unigene0008403), SAD (Unigene0012943), DHLAT (Unigene0014324), α-CT (Unigene0022151) and KAS II (Unigene0024371) that were significantly up-regulated in the final stage of seed development. A total of 66 unigenes encoding key enzymes involved in the synthesis of triacylglycerols (TAGs) were found, along with seven unigenes encoding PDCT (Unigene0000909), LPCAT (Unigene0007846), Oleosin3 (Unigene0010027), PDAT1 (Unigene0016056), GPDH (Unigene0022660), FAD2 (Unigene0037808) and FAD3 (Unigene0044238); these also proved to be up-regulated in the final stage of seed development.

## Introduction

Sacha inchi (*Plukenetia volubilis* L.), commonly known as Inca peanut, Inca inchi, mountain peanut or wild peanut, is native to Amazon rainforests where it grows in warm climates at altitudes between 200 and 1500 m above sea level^1,2^. This perennial species is from the Euphorbiaceae family and is an oleaginous, semi-ligneous vine to shrub like plant growing to heights of up to 2 m. From as young as age 1 year it produces green lobed fruits (with 4–7 points), and when ripe these fruits contain oval, dark-brown seeds of 1.5–2 cm^3,4^.

The species is now attracting commercial interest as its seeds contain 35–60% edible oil (by dry weight)—a content comparable to that of sunflower seeds and peanuts—as well as being high in protein (approximately 27% or more by dry weight) and rich in vitamins A and E^5,6^. In contrast to the oils from the more traditional oil seed species such as sunflower and peanut, that from sacha inchi contains large amounts of polyunsaturated fatty acids (PUFAs can account for up to 80% or more of its seeds’ total fatty acids) with linoleic acid (18:2 cis-Δ9,12; LA) and α-linolenic acid (18:3 cis-Δ9,12,15; ALA) comprising up to 80% or more of these PUFAs. As well as being rich in ALA, the ratio of omega-6 to omega-3 fatty acids (FAs) in its oil is approximately 7:10^7–9^. Traditional oil seed crops such as soybean, peanut, maize, and canola yield oils with comparatively low ALA contents (below 10%) and higher omega-6 to omega-3 ratios (approximately 1), which is thought to be a major contributor to cardiovascular disease^10^. The aforementioned characteristics of sacha inchi’s seed oil set it apart as being superior from other oil seed species; its oil is considered one of the healthiest of all vegetable oils^11,12^. The key fatty acids found in sacha inchi seed oil—ALA (an omega-3 fatty acid) and LA (an omega-6 fatty acid)—are essential for humans yet rarely found in such favourable proportions in a single source.

Even though sacha inchi seeds are now known to have good economic potential on account of their oil and other constituents, this recognition has come only recently, and little research has yet been done on the physiological mechanisms responsible for lipid metabolism and regulation of related unigene expression in sacha inchi seeds^7,13^. Previous studies in other oil seed species have established that biosynthesis and accumulation of plant oils are achieved through a series of enzyme reactions along the pathways of fatty acid (FA) and triacylglycerol (TAG) biosynthesis^14^. And, studies of various other oleaginous seed producing species have found that the pathways of FA biosynthesis in such plants occur primarily in chloroplasts whilst those for TAG biosynthesis occur primarily in endoplasmic reticulum^15–17^. TAGs are esters of glycerol composed of three fatty acids esterified with a glycerol backbone. Generally, TAGs are the major fats accumulated specifically in endosperms and embryos of oil seeds^15,18^.

One of the few studies undertaken on oil development in sacha inchi seed to date examined molecular mechanisms responsible for biosynthesis of unsaturated fatty acids during two nominal seed development stages: an initial stage, and a later stage deemed by the authors as the fast oil accumulation stage^7^. However, subsequent studies have indicated that more than two distinct stages can be identified in growth and development of sacha inchi seeds, with respect to variations in fatty acid contents^9^. Most recently, Liu et al.^19^ identified five distinct stages based on lipid metabolite content: an initial formation stage (SI-1), an early development stage (SI-2), a middle stage (SI-3), a later stage (SI-4) and a maturation stage (SI-5). The key changes found through these stages included that ALA content decreased by 41.4% from SI-1 to SI-2 before increasing steadily through SI-3 and SI-4 and finally showing a 9.86-fold increase from SI-4 to SI-5. Meanwhile, linoleic acid content reached its lowest level in SI-2, and content of this fatty acid then gradually increased to SI-5, including a 3.10-fold increase from SI-4 to SI-5.

This current study was initiated to better understand the molecular mechanisms underlying development of fatty acid accumulation in sacha inchi seeds through the process of their development. To do this, the five aforementioned stages of sacha inchi seed development were used as a basis for identifying candidate genes associated with oil accumulation and fatty acid production during development of its seeds. Comparative de novo transcriptomic analyses of seeds from each of the five stages was carried out using high-throughput Illumina RNA-sequencing. Subsequent assembling and annotation of transcriptome sequences and unigene expression profiles was undertaken to identify enzymes and related regulatory unigenes involved in oil biosynthesis and accumulation in this species.

## Results

### Transcriptome sequencing and de novo assembly

Five cDNA libraries were constructed, one from each of the five recognized stages of sacha inchi seed development (i.e. stages SI-1, SI-2, SI-3, SI-4 and SI-5), and sequenced by Illumina HiSeq 4000 system. There was a total of 751.25 million 150-bp paired-end reads generated from these five libraries (average of 50.08 million), encompassing 109.86 Gb of sequence data (average of 7.32 Gb) (Table 1). After stringent quality assessment and data filtering, a total of 739.72 million high-quality reads (average of 49.31 million) were selected for further analysis. The Q20 percentage reached more than 98%, and the GC content of the five libraries ranged from 43.17 to 44.97%.

**Table 1:** Summary statistics of clean transcriptome sequencing data for the five stages of sacha inchi seed development.

Sample	Development stage (time period)	Total reads	Total nucleotides (bp)	High-quality reads (%)	Q20 (%)	GC (%)	Adapter (%)
SI-1-1	SI-1 (5–10 DAF)	52,987,294	7,735,958,631	52,136,962 (98.40%)	7,627,276,369 (98.60%)	43.20	182,260 (0.34%)
SI-1-2		51,013,880	7,437,747,908	50,114,654 (98.24%)	7,343,508,300 (98.73%)	43.18	179,126 (0.35%)
SI-1-3		54,164,718	7,927,830,910	53,361,226 (98.52%)	7,845,113,079 (98.96%)	43.21	193,936 (0.36%)
SI-2-1	SI-2 (15–30 DAF)	50,952,584	7,463,224,006	50,203,062 (98.53%)	7,384,519,968 (98.95%)	43.54	180,682 (0.35%)
SI-2-2		54,269,786	7,921,184,920	53,331,880 (98.27%)	7,833,525,933 (98.89%)	43.26	193,690 (0.36%)
SI-2-3		50,733,028	7,422,519,528	49,941,442 (98.44%)	7,345,510,339 (98.96%)	43.27	182,568 (0.36%)
SI-3-1	SI-3 (35–50 DAF)	50,609,808	7,390,164,229	49,784,910 (98.37%)	7,300,825,915 (98.79%)	43.53	184,462 (0.36%)
SI-3-2		49,902,984	7,287,587,733	49,076,322 (98.34%)	7,198,489,164 (98.78%)	44.11	187,354 (0.38%)
SI-3-3		48,545,556	7,106,526,217	47,847,274 (98.56%)	7,023,633,037 (98.83%)	43.38	172,938 (0.36%)
SI-4-1	SI-4 (55–90 DAF)	45,890,158	6,714,896,703	45,230,004 (98.56%)	6,634,930,706 (98.81%)	43.17	161,592 (0.35%)
SI-4-2		48,689,108	7,136,472,622	48,032,904 (98.65%)	7,055,199,856 (98.86%)	43.36	179,518 (0.37%)
SI-4-3		58,697,444	8,595,171,247	57,882,956 (98.61%)	8,495,839,669 (98.84%)	43.33	211,296 (0.36%)
SI-5-1	SI-5 (95–120 DAF)	43,306,104	6,332,416,755	42,657,532 (98.50%)	6,255,440,276 (98.78%)	44.06	147,612 (0.34%)
SI-5-2		37,540,872	5,495,306,999	37,011,534 (98.59%)	5,430,666,333 (98.82%)	44.97	108,840 (0.29%)
SI-5-3		53,946,542	7,879,167,277	53,104,334 (98.44%)	7,782,235,023 (98.77%)	44.24	177,132 (0.33%)

*DAF *days after flowering, *Q20  *percentage of bases with quality values larger than 20, *GC *guanidine and cytosine nucleotides as a proportion of all nucleotides (expressed as a percentage).

**Figure 1:**
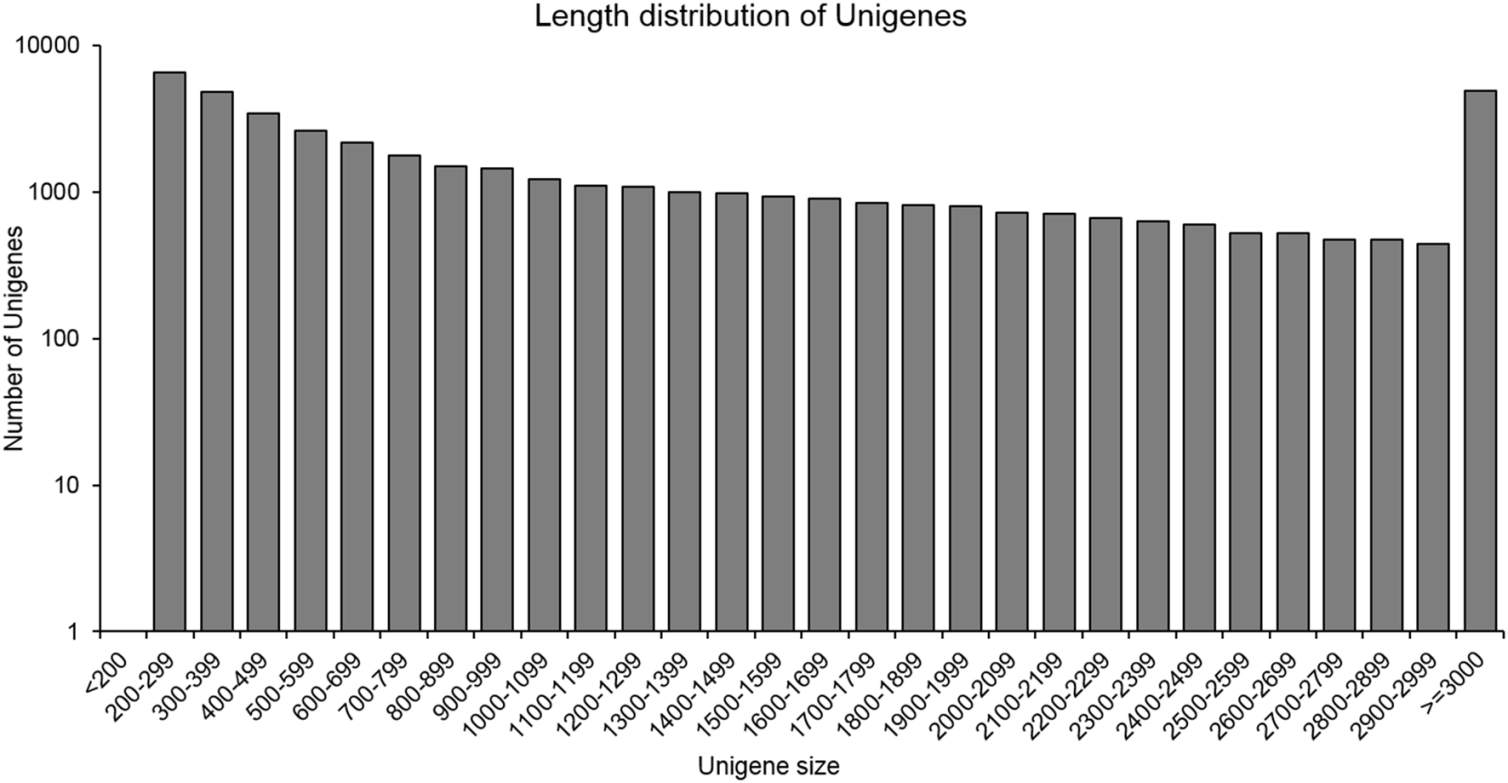
The length distribution of the unigenes obtained from sacha inchi seeds by RNA-seq.

The high-quality reads obtained from the transcriptome libraries of the five stages were pooled and assembled using Trinity 2.8.6 software (Table 2), with 60.67 M bases being assembled and 44,797 unigenes were obtained with a mean length of 1,354 base pairs (bp) and an N50 of 2,299 bp. Of these unigenes, 39.09% of the nucleotides were guanidine and cytosine. The length distribution showed that 17,490 unigenes (39.05%) were shorter than 600 bp in length and 20,379 (45.49%) were longer than 1000 bp (Fig. 1). Results of BUSCO analysis showed that the assembly retrieved 88.40% of the conserved single copy orthologous genes, including 73.13% of the complete (C) and 15.27% of fragment genes (F). These latter results indicate that most orthologs were accurately identified as complete single copy (C:1053 [S:925, D:128], F:220, M:167, N:1440), whilst the high completeness (C) and low percentages of both fragments (F) and missing sequences (M) reveal that the transcriptome assembly had good representation and provided a good de novo assembled transcriptome of sacha inchi seed.

**Table 2:** Parameters from the assembly of transcriptome reads from sacha inchi seeds.

Parameter	Value
Gene number	44,797
GC percentage (%)	39.09
N50	2,299
Maximum length	17,015
Minimum length	201
Average length	1,345
Total assembled bases	60.67 million

### Sequence annotation

**Figure 2:**
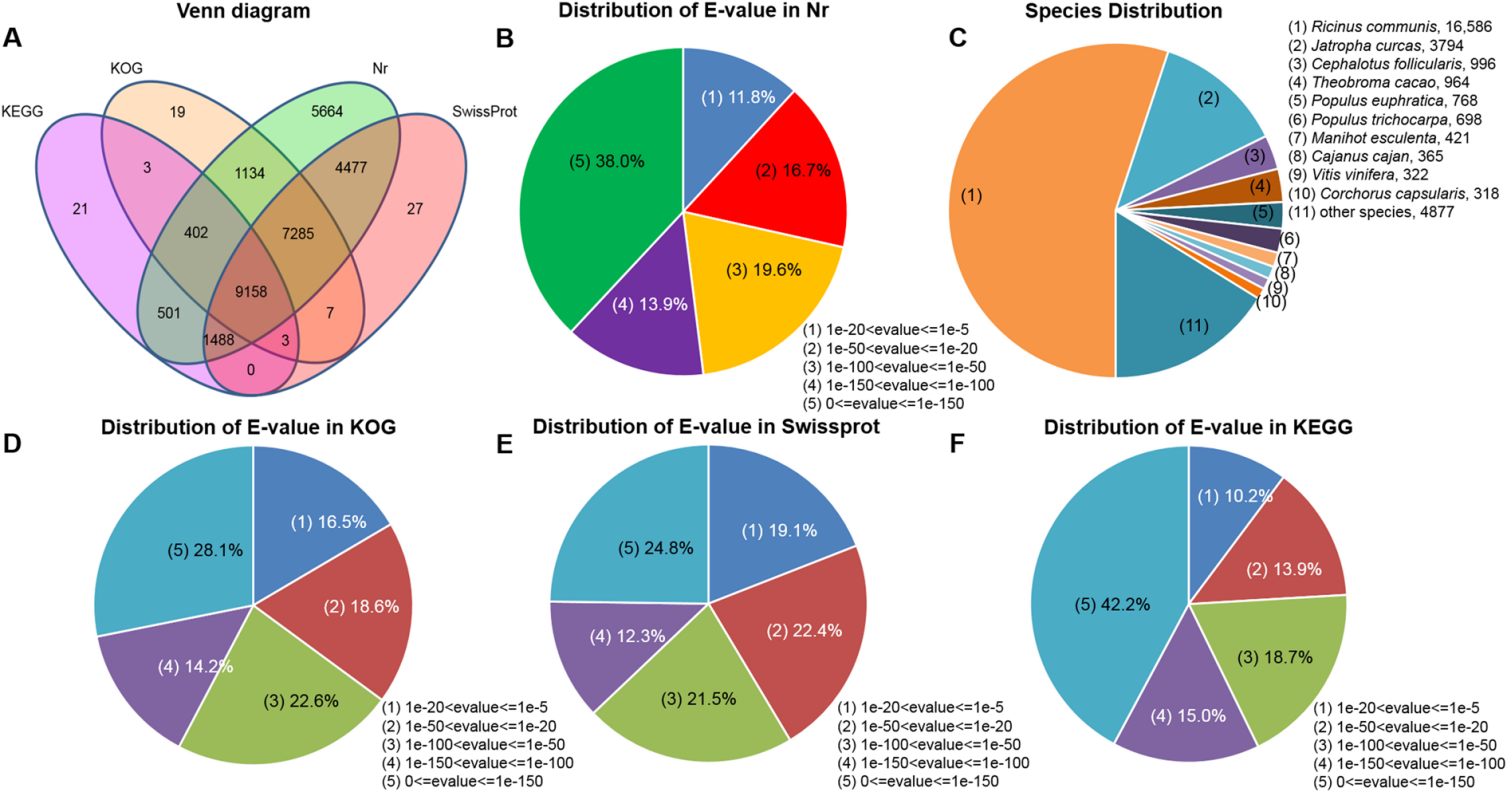
Characteristics of homology searches of sacha inchi unigenes: (**A**) Venn diagram of number of unigenes annotated by BLASTx with a cut-off value of 1e−5 against protein databases, with encircled numbers indicating the number of unigenes annotated by single or multiple databases; (**B**–**F**) E-value distribution of the top BLASTx hits against the Nr, KOG, Swissprot and KEGG databases respectively; and, (**C**) number of unigenes matching the 10 top species using BLASTx in the Nr database.

Using ESTScan 2.0b software^20^ with a cut-off E-value of 1e−5, 44,797 unigenes were subject to BLASTx searches against the NCBI non-redundant (Nr), Swiss-Prot, Kyoto Encyclopedia of Genes and Genomes (KEGG) and Cluster of Orthologous Groups of Protein (COG/KOG) databases (Supplementary Table S2). To annotate these unigenes, a homology search using BLASTx program (E-value cut-off 1e−5) revealed that 67.2% (30,109) could be annotated in the NCBI Nr database, while 50.1% (22,445) were annotated using the Swiss-Prot database. In addition, 25.8% (11,576) and 40.2% (18,011) of unigenes could be annotated according to the KEGG and COG/KOG database, respectively. Altogether, 30,189 (67.4%) unigenes were successfully annotated in the four public databases (Fig. 2A).

Of the unigenes that could be annotated using the NCBI Nr database, 88.2% showed strong homology (E-value < 1e−20) to available plant sequences, with 71.5% of the E-values ranging from 0 to 1e−50 (Fig. 2B). Similarly, the E-value distribution of the annotated unigenes in KOG, Swissprot, KEGG databases (Fig. 2D–F) showed that there were higher levels homology (< 1e−20) to available plant sequences (80.9%, 89.8% and 83.5%, respectively).

As shown in Fig. 2C, 25,232 (83.8%) of these unigenes were annotated to 10 top-hit species using the NCBI Nr database. It was noteworthy that 16,586 (55.1%) of these unigenes were annotated to *Ricinus communis* and 3,794 (12.6%) to *Jatropha curcas*; both these species and sacha inchi belong to the Euphorbiaceae family.

### GO, KOG and KEGG classification

**Figure 3:**
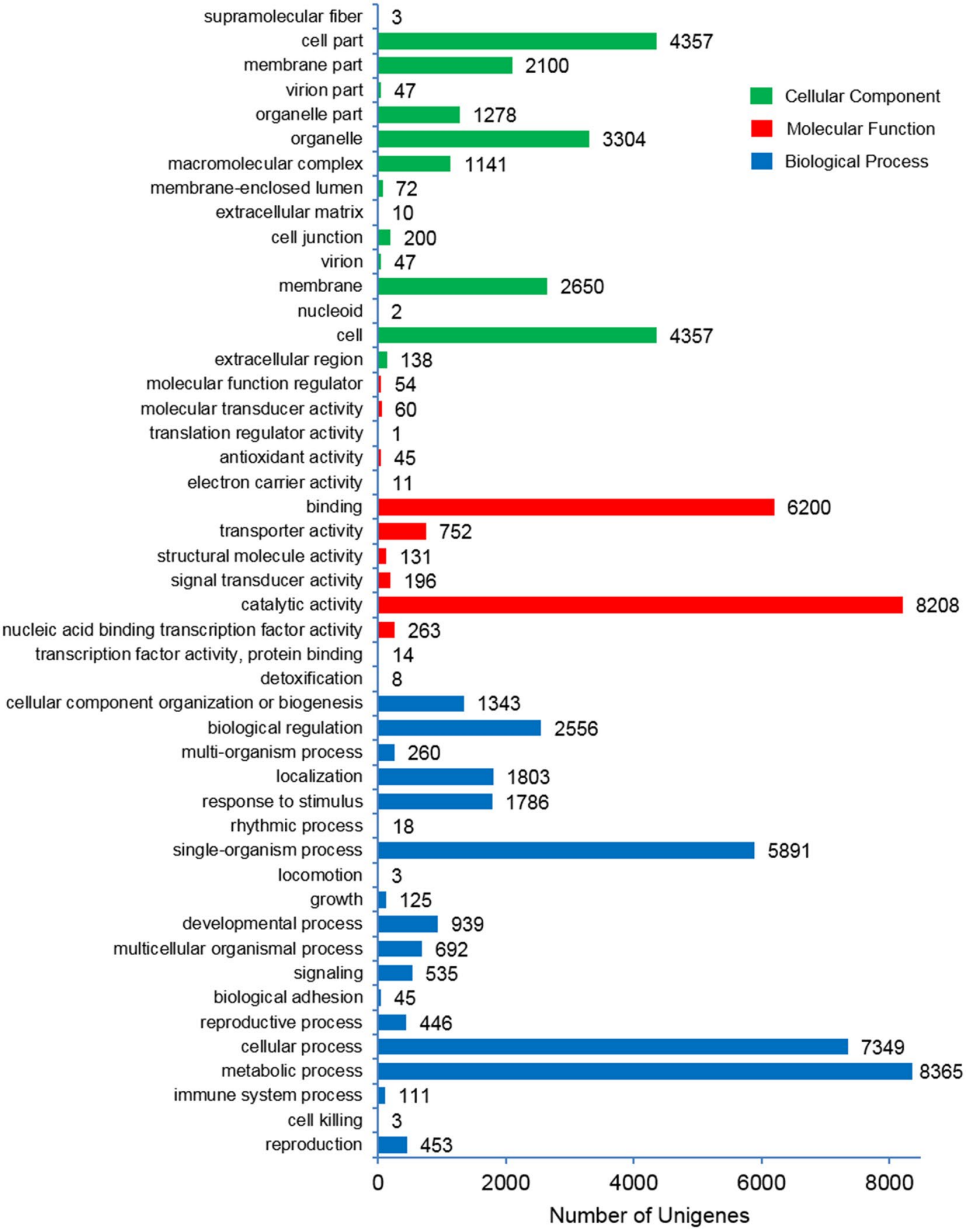
Histogram of Gene Ontology (GO) assignments for transcriptome sequences of sacha inchi seeds; the unigenes corresponded to three main categories: biological processes (blue bars); cellular components (green bars); and, molecular functions (red bars), with the length of each bar (and numbers at the tail of each bar) indicating the number of unigenes in each sub-category.

Using Blast2GO 5.2.5 software, Gene Ontology (GO) analyses of unigenes were performed to classify the functions of the sacha inchi unigenes, based on high-score BLAST matches in the NCBI Nr database. From these analyses, 14,453 unigenes were classified into three main GO categories and 47 sub-categories (Fig. 3). According to this GO annotation, the biological process categories included metabolic processes (8365 unigenes; 57.9% of the total), cellular processes (7349 unigenes; 50.8%), single-organism processes (5891 unigenes; 40.8%), biological regulation (2556 unigenes; 17.7%), localization (1803 unigenes; 12.5%), response to stimuli (1786 unigenes; 12.4%), cellular component organization or biogenesis (1343 unigenes; 9.3%) and other sub-categories (3638 unigenes; 25.2%). Additionally, cellular component categories included cells (4357 unigenes; 30.1% of the total), cell parts (4357 unigenes; 30.1%), organelles (3,304 unigenes; 22.9%), membranes (2650 unigenes; 18.3%), membrane parts (2100 unigenes; 14.5%), organelle part (1278 unigenes; 8.8%), macromolecular complexes (1141 unigenes, 7.9%) and other sub-categories (519 unigenes; 3.6%). Molecular function categories included catalytic activity (8208 unigenes; 56.8% of the total), binding (6200 unigenes; 42.9%), transporter activity (752 unigenes; 5.2%) and other sub-categories (775 unigenes; 5.4%).

**Figure 4:**
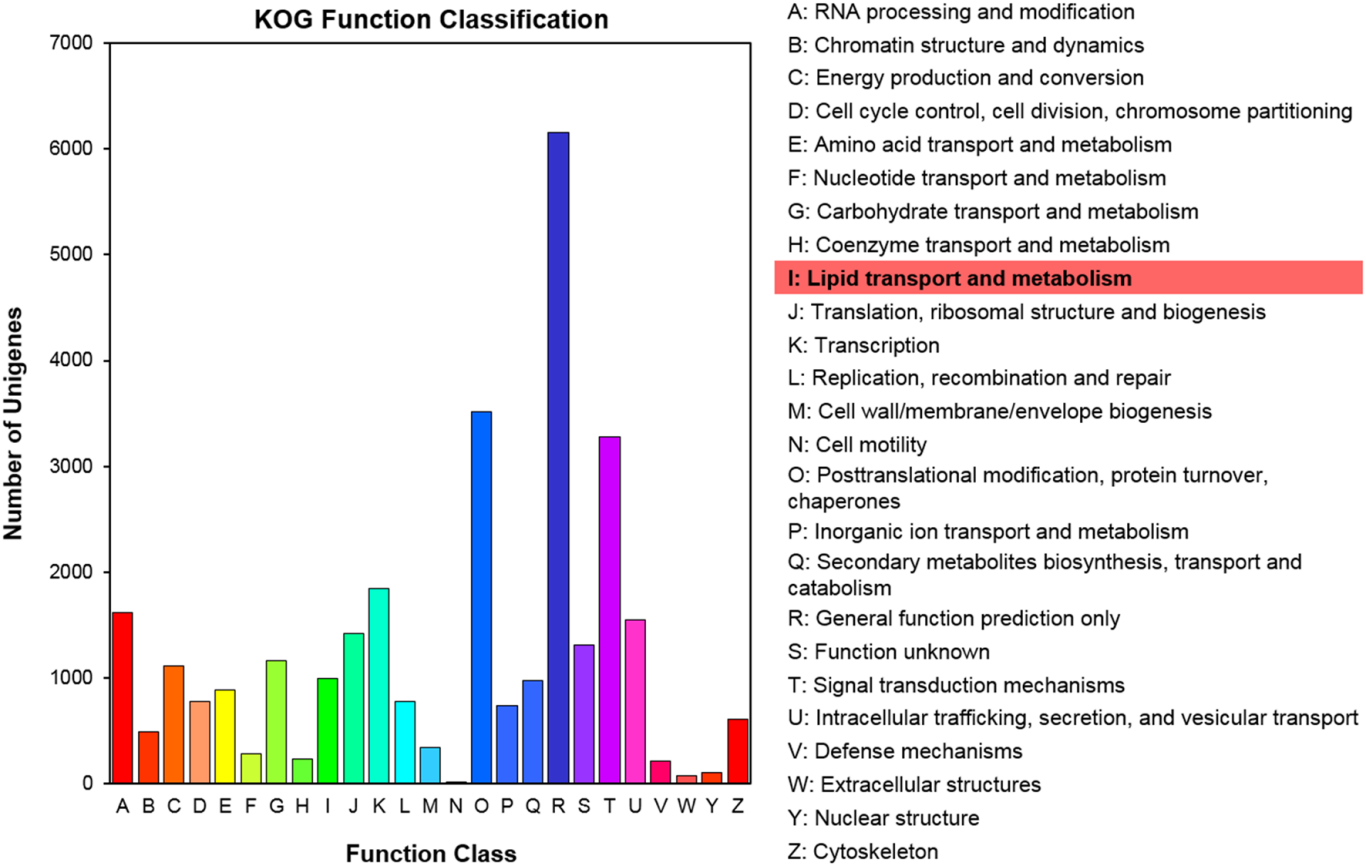
EuKaryotic Ortholog Group (KOG) functional classification for the transcriptome sequences of sacha inchi unigenes classified into 25 KOG categories (listed as A to Z in the key on the right of the figure). The y-axis indicates the number of unigenes in each KOG category.

To compare predicted and known proteins encoded by these unigenes to all known eukaryotic genomes, the COG/KOG database was interrogated. This analysis assigned 18,011 unigenes into 25 KOG categories (Fig. 4): including the categories of general function prediction (6151 unigenes; 34.2% of the total), post-translational modification, protein turnover and chaperones (3513 unigenes; 19.5%), signal transduction mechanisms (3277 unigenes; 18.2%), transcription (1848 unigenes; 10.3%), RNA processing and modification (1620 unigenes; 9.0%), intracellular trafficking, secretion and vesicular transport (1547 unigenes; 8.6%) and lipid transport and metabolism (990 unigenes; 5.5%). This latter category mainly related to the lipid biosynthesis and accumulation during sacha inchi seed growth. However, categories with no concrete assignment, such as function unknown (1314 unigenes; 7.3% of the total) and general function prediction (6151 unigenes; 34.2%) accounted for a large proportion of total transcripts.

**Figure 5:**
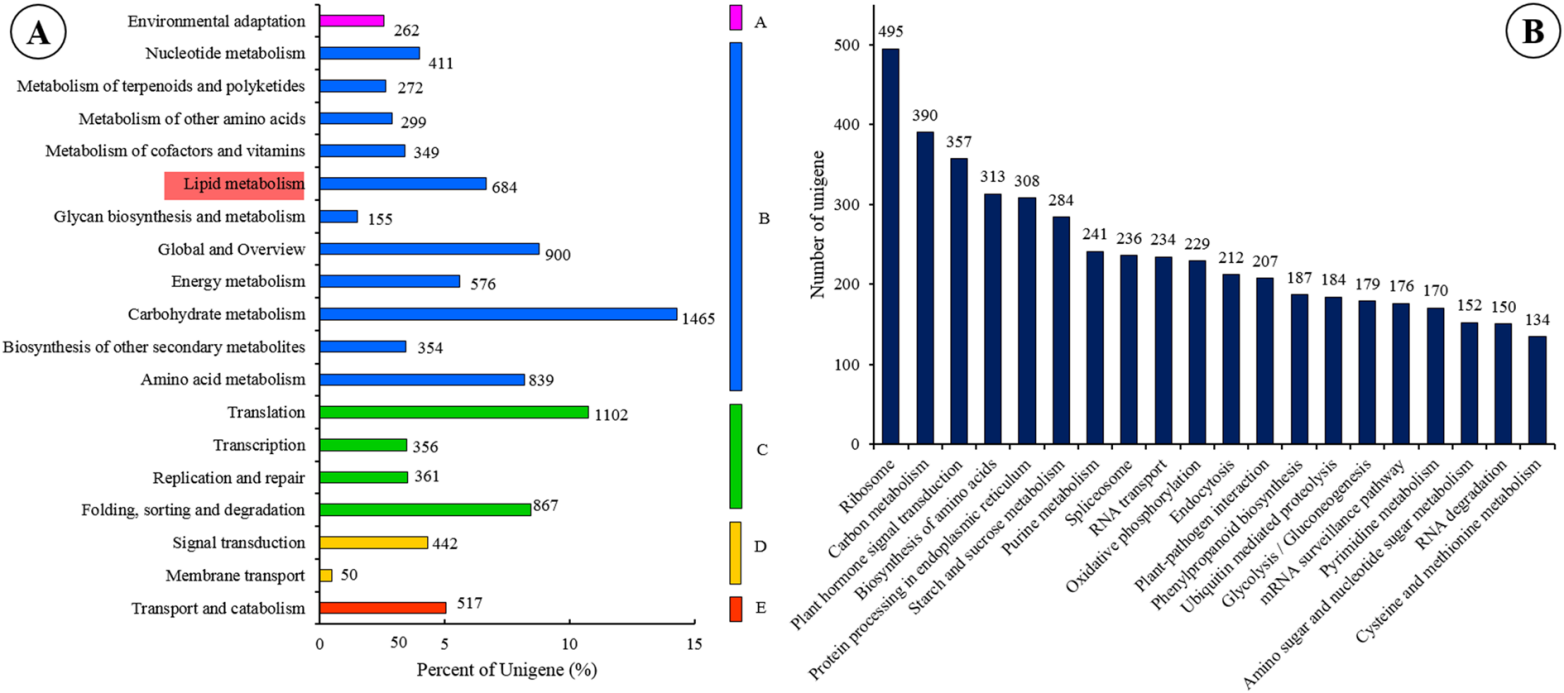
Kyoto Encyclopedia of Genes and Genomes (KEGG) annotation for the transcriptome sequences of sacha inchi: (**A**) as unigenes classified into five categories on the basis of the KEGG database, including A (pink colour): Organismal System; B (blue colour): Metabolism; C (green colour): Genetic Information Processing; D (yellow colour): Environmental Information Processing; E (red colour): Cellular Processes, with the x-axis indicating the percentage of unigenes in each category; and, (**B**) the 20 pathways with the highest numbers of annotated and enriched unigenes are shown along the x-axis with the y-axis indicating the number of unigenes in each pathway.

KEGG pathway-based analysis assigned 11,576 unigenes to a total of 128 KEGG biochemical pathways, with the majority of these unigenes shown to be involved in metabolic pathways (Fig. 5A). These pathways included carbohydrate metabolism (14.3% of these 11,576 unigenes), amino acid metabolism (8.2%) and lipid metabolism (6.7%), some of which are linked to changes in oil content and composition that take place during sacha inchi seed development. For example, lipid metabolism processes involve the interaction and degradation of lipids, and the pathways involved in this included glycerophospholipid metabolism (ko00564; 115 unigenes), glycerolipid metabolism (ko00561; 86 unigenes), α-linolenic acid metabolism (ko00592; 67 unigenes), fatty acid degradation (ko00071; 67 unigenes), fatty acid biosynthesis (ko00061; 65 unigenes), biosynthesis of unsaturated fatty acids (ko01040; 51 unigenes), sphingolipid metabolism (ko00600; 45 unigenes), steroid biosynthesis (ko00100; 42 unigenes), fatty acid elongation (ko00062; 40 unigenes), cutin, suberine and wax biosynthesis (ko00073; 25 unigenes), linoleic acid metabolism (ko00591; 21 unigenes), arachidonic acid metabolism (ko00590; 18 unigenes) and synthesis and degradation of ketone bodies (ko00072; 7 unigenes). Among these, fatty acid degradation (ko00071), fatty acid biosynthesis (ko00061), biosynthesis of unsaturated fatty acids (ko01040), fatty acid elongation (ko00062), cutin, suberine and wax biosynthesis (ko00073) and linoleic acid metabolism (ko00591) that were involved in the KEGG pathway related to the fatty acid metabolites showed significant differences through the five different stages of sacha inchi seed development^19^.

The fatty acid metabolic pathway revealed through the KEGG pathway analysis belonged to the global and overview sub-category and this includes catabolic processes that generate energy and primary metabolites from fatty acids, and anabolic processes that create biologically important molecules from fatty acids and other sources. The identification of these lipid unigenes provides critical clues to assist analyses of key functional unigenes involved in lipid body formation and unsaturated fatty acid biosynthesis in sacha inchi seeds.

The top 20 pathways, based on numbers of unigenes annotated to them by the KEGG analysis, are represented in Fig. 5B. That with the highest unigene representation was the ribosome pathway (ko03010) with 495 unigenes, followed by a carbon metabolism pathway (ko01200; 390 unigenes), plant hormone signal transduction pathway (ko04075; 357 unigenes), amino acid biosynthesis pathway (ko01230; 313 unigenes), protein processing in endoplasmic reticulum pathway (ko04141; 308 unigenes), starch and sucrose metabolism pathway (ko00500; 284 unigenes), purine metabolism pathway (ko00230; 241 unigenes) and spliceosome pathway (ko03040; 236 unigenes).

### Analysis of DEGs at the five developmental stages

**Figure 6:**
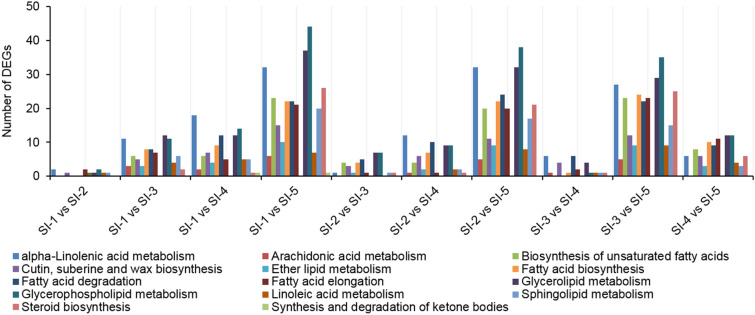
KEGG classification of differentially expressed lipid genes in 10 pairwise comparisons among the five seed development stages in sacha inchi.

The DEG analysis carried out to provide insights into patterns of significantly changes in gene actions during the five seed development stages revealed that 20,446 DEGs in KEGG pathways to be the noteworthy among the top 14 KEGG pathways related to lipid metabolism, based on pairwise comparisons among the five seed development stages (Fig. 6). The number of DEGs in pathways of α-linolenic acid metabolism (ko00592), Glycerophospholipid metabolism (ko00564) and Glycerolipid metabolism (ko00561) were the most abundant. The comparable group of SI-1 vs SI-5 had the largest number of DEGs, and next were SI-2 vs SI-5 and SI-3 vs SI-5 while the lowest numbers of DEGs were observed in the comparisons of SI-1 vs SI-2 and SI-3 vs SI-4.

### Identification and characterization of genes involved in ALA biosynthesis

**Figure 7:**
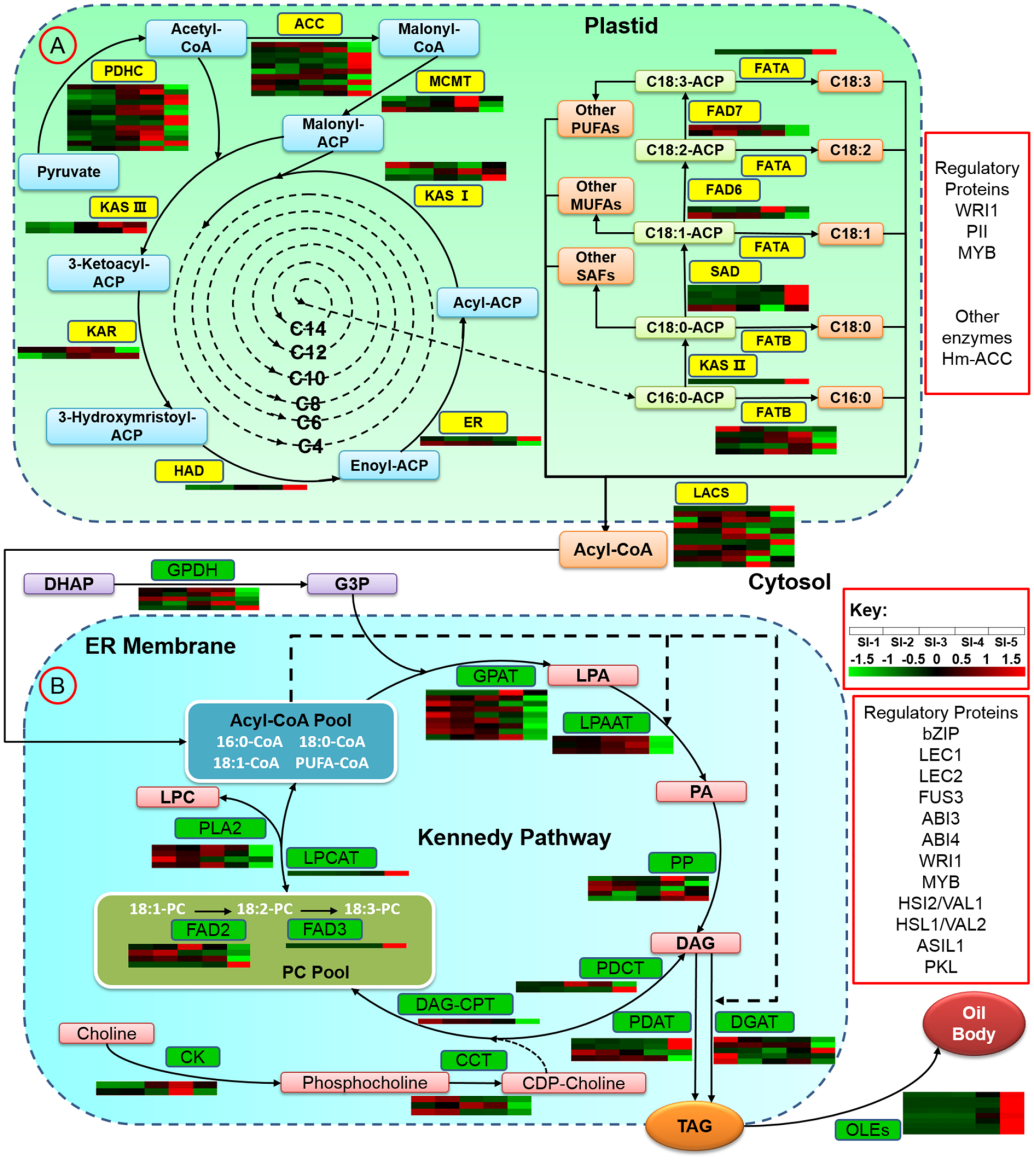
Identification of genes in the pathways of fatty acid synthesis (**A**) and triacylglycerol synthesis (**B**), based on the transcriptome of *P. volubulis* seeds—the icons below each enzyme ID show the expression patterns of the transcripts through each of the five seed developmental stages, with the color scale indicating the level of expression of unigenes: deeper red indicates higher expression while greener colours represent lower expressions. Note: expression patterns are shown only for unigenes that were found to have RPKM values of ≥ 2.

Based on the assembly and annotation of the *P. volubilis* seed transcriptome, a total of 86 transcripts were identified as candidate genes for 15 enzymes of ALA biosynthesis (Table 3). To better understand the processes involved, a schematic map of the role of these enzymes in the was produced (Fig. 7A).

**Table 3:** Key enzymes related to ALA biosynthesis and TAG assembly identified by annotation of the *P. volubilis* seed transcriptome.

Symbol	Enzymes	EC number	Unigene number
**ALA biosynthesis**			
PDHC	Pyruvate dehydrogenase complex (pyruvate dehydrogenase or E1; dihydrolipoamide acetyltransferase or E2; dihydrolipoamide dehydrogenase or E3)	1.2.4.1	6
		2.3.1.12	6
		1.8.1.4	5
ACC	Acetyl-CoA carboxylase	6.4.1.2	15
MCMT	Malonyl-CoA: ACP malonyltransacylase	2.3.1.39	3
KAS I	3-Ketoacyl-ACP synthase I	2.3.1.41	4
KAS II	4-Ketoacyl-ACP synthase II	2.3.1.179	1
KAS III	5-Ketoacyl-ACP synthase III	2.3.1.180	3
KAR	3-Ketoacyl-ACP reductase	1.1.1.100	4
HAD	3-Hydroxyacyl-ACP dehydratase	4.2.1.59	1
ER	Enoyl-ACP reductase	1.3.1.9	2
SAD	Acyl-ACP desaturase	1.14.19.2	6
FATB	Palmitoyl-acyl carrier protein thioesterase	3.1.2.14	10
FATA	Oleoyl-acyl carrier protein thioesterase	3.1.2.14	1
FAD6	Oleate desaturase(chloroplast)	1.14.19.39	3
FAD7	Linoleate desaturase (chloroplast)	1.14.19.3	2
LACS	Long-chain acyl-CoA synthetase	6.2.1.3	14
**TAG assembly**			
GPDH	Glycerol-3-phosphate dehydrogenase	1.1.1.8	5
GPAT	Glycerol-3-phosphate acyltransferase	2.3.1.15	11
LPAAT	Lysophosphatidate acyltransferase	2.3.1.51	4
PP	Phosphatidate phosphatase	3.1.3.4	5
DGAT	Diacylglycerol acyltransferase	2.3.1.20	6
PDAT	Phospholipid: diacylglycerol acyltransferase	2.3.1.43	7
PDCT	Phosphatidylcholine: diacylglycerol cholinephosphotransferase	2.7.8.2	2
CK	Choline kinase	2.7.1.32	2
DAG-CPT	Diacylglycerol cholinephosphotransferase	2.7.8.2	2
FAD2	Oleate desaturase (endoplasmic reticulum)	1.14.19.39	4
FAD3	Linoleate desaturase (endoplasmic reticulum)	1.14.19.3	1
CCT	Choline-phosphate cytidylyltransferase	2.7.7.15	3
LPCAT	2-lysophosphatidylcholine acyltransferase	2.3.1.23	1
PLA2	Phospholipase A2	3.1.1.4	5
OLEs	Oleosins, steroleosins; caleosins	–	8

The initial and acyl chain elongation step of de novo ALA biosynthesis is known to use acetyl-CoA. In sacha inchi seeds, the most straightforward pathway that rapidly generates acetyl-CoA to maintain the pool is through the action of the 17 unigenes encoding the enzymes of plastidial pyruvate dehydrogenase complex (PDHC) subunits (six for pyruvate dehydrogenase or E1, PDH, EC 1.2.4.1; six for dihydrolipoamide acetyltransferase or E2, DHLAT, EC 2.3.1.12; and five for dihydrolipoamide dehydrogenase or E3, LPD, EC 1.8.1.4). Acetyl-CoA is initially catalyzed by 15 unigenes encoding acetyl-CoA carboxylase (ACC; EC 6.4.1.2; one for biotin-carboxylase, BC; five for biotin-carboxyl carrier protein, BCCP; six for α-CT and three for β-CT of carboxyltransferase) subunits to form malonyl-CoA. Two unigenes encoding the enzyme malonyl-CoA: ACP malonyltransacylase (MCMT, EC 2.3.1.39) catalyzes the conversion of malonyl-CoA to malonyl-ACP, which is the primary substrate for a subsequent cycle of condensation reactions^21^. Eight unigenes encoding 3-ketoacyl-ACP synthase (KAS; four for KASI, one for KASII, and three for KASIII, respectively), and seven unigenes encoding other components of FA synthase (four for 3-ketoacyl-ACP reductase, KAR, EC 1.1.1.100; one for 3-hydroxyacyl-ACP dehydratase, HAD, EC 4.2.1.59; and two for enoyl-ACP reductase, ER, EC 1.3.1.9).

The condensation reaction cycle was repeated four times and then four unigenes encoding 3-ketoacyl-ACP synthase I (KAS I, EC 2.3.1.41) were used for the elongation from C4 to C16. But, the reaction from C14 to C18 in the ALA biosynthesis pathway is catalyzed by the Unigene0024371 encoding 3-ketoacyl-ACP synthase II (KAS II, EC 2.3.1.179). Through these processes, the acetyl-CoA turns into C16:0-ACP or C18:0-ACP, and then the six unigenes encoding enzyme acyl-ACP desaturase (SAD, EC 1.14.19.1) remove two hydrogen atoms from C18:0-ACP to form C18:1-ACP. Meanwhile, one unigene was found to encode fatty acyl-ACP thioesterase A (FATB, EC 3.1.2.14), and this releases C18:1-ACP to C18:1. A total of three unigenes were introduced to encode the enzymes of fatty acid desaturase 6 (FAD6, EC 1.14.19.22) that can desaturate oleic acid (OA, C18:1^∆9^) to generate linoleic acid (LA, C18:2^∆9,12^)^22^. Three other unigenes found encoded the enzyme of fatty acid desaturase 7 (FAD7, EC:1.14.19.35), which is known to act on the omega-6 fatty acid LA to catalyze the biosynthesis of α-linolenic acid (ALA, C18:2^∆9,12,15^) from LA in *P. volubilis* seed^12^. These fatty acids are ultimately activated to CoA esters by a long-chain acyl-coenzyme A synthetases (LACSs) and exported to the endoplasmic reticulum (ER) or possibly enter phosphatidylcholine (PC) at the plastid envelope by the action of lysophosphatidylcholine acyltransferase (LPCAT; EC 2.3.1.23)^14,23^.

In the ALA biosynthesis pathway, the RPKM value showed that all of the unigenes encoding MCMT, HAD, KAS II and FATA showed significant up-regulation in the SI-5 stage compared with other stages (Fig. 7A and Supplementary Table S3). Transcriptional profiling in sacha inchi seeds identified twenty-eight unigenes with higher transcript levels at the seed development stage SI-5. Among them, three (Unigene0022149, Unigene0022151 and Unigene0033318) encoding α-CT, Unigene0003906 encoding BC, two (Unigenes0011273 and Unigene0019543) encoding BCCP, Unigene0012093 encoding ER, Unigene0023136 encoding FATB, Unigene0016529 encoding the enzyme KASI, Unigene0024371 encoding the enzyme KASII, three (Unigene0012941, 0012942, 0012943) encoding SAD and two (Unigene0000156 and Unigene0022603) encoding LACS showed significant up-regulation in the SI-5 stage. However, all of the unigenes encoding FAD6 showed significant down-regulation in the SI-5 stage compared with the other earlier stages (Fig. 7A and Supplementary Table S3).

### Expression analysis of TAG assembly genes at different development stages of *P. volubilis* seed

Neutral lipids in oleaginous seeds are mainly stored in the form of TAGs, which are themselves composed of fatty acid residues and glycerol. TAG biosynthesis occurs at the ER and probably also involves reactions at the lipid body^24^. In plant seeds, FA production by plastids could limit TAG accumulation, so increasing flux through FA biosynthesis may perhaps have the greatest influence on the amount of TAG assembled in plant seeds^25^. In the process of TAG assembly, 66 transcripts were identified as candidate unigenes for 15 enzymes (Table 3).

As shown in the Fig. 7B, glycerol-3-phosphate (G3P) which is formed by the reduction of dihydroxyacetonephosphate (DHAP) is acylated by 11 of the unigenes encoding glycerol-3-phosphate acyltransferase (GPAT; EC 2.3.1.15) to yield 2-lysophosphatidic acid (LPA), followed by further acylation by four unigenes encoding lysophosphatidate acyltransferase (LPAAT; EC 2.3.1.51) to produce phosphatidic acid. This is followed by dephosphorylation catalyzed by five unigenes encoding phosphatidate phosphatase (PP; EC 3.1.3.4) to release diacylglycerol (DAG), which is acylated by six unigenes encoding diacylglycerol acyltransferase (DGAT; EC 2.3.1.20) to form TAG. Also, DAG can be catalyzed to TAG by seven unigenes encoding phospholipid: diacylglycerol acyltransferase (PDAT; EC 2.3.1.43) using PC as the acyl donor. The synthesis of TAG by PDAT is dependent on Unigene0007846 encoding the enzyme of LPCAT activity for the regeneration of PC from 2-lysophosphatidylcholine (LPC). Additionally, it is well known that other enzymes are important for synthesis of TAGs in plants such as two unigenes encoding phosphatidylcholine: diacylglycerol cholinephosphotransferase (PDCT; EC 2.7.8.2), that catalyzes the exchanges phosphocholine between DAG and PC, as well as two unigenes encoding diacylglycerol cholinephosphotransferase (DAG-CPT; EC 2.7.8.2) which catalyzes the reverse reaction^26^. Free FAs released by the hydrolysis of phospholipids by five unigenes encoding the enzyme of Phospholipase A2 (PLA2, EC 3.1.1.4) enter the acyl-CoA pool for TAG synthesis.

Once TAGs are synthesized, layers of phospholipids and protein steroleosins, oleosins and caleosins will surround these TAGs to form structures referred to as lipid droplets or oil bodies^27,28^. According to the analyses of the expression of the unigenes encoding these related enzymes in sacha inchi seeds, eight unigenes encoding OLEs showed significant differences among the five stages of seed development observed in this study. Generally in oilseeds, FAD3 is responsible for the production of the majority of omega-3 fatty acids and ALA in particular^29^, and in sacha inchi the expression pattern of Unigene0044238 coincided with the ALA accumulation pattern and revealed that the expression profile of FAD3 gene is correlated with ALA synthesis in seeds of this species. Similarly, Unigene0007846 encoding LPACT, and eight unigenes encoding the enzyme of oleosin along with the previously reported Unigene0037808 encoding FAD2^19^ showed more upregulation at the SI-5 stage compared with the four earlier stages (Fig. 7B). The expression of OLEs unigenes is closely associated with oil accumulation in developing seeds^7,30^. The eight unigenes encoding OLEs were highly expressed at SI-5 stage. However, three unigenes encoding FAD2, four unigenes encoding LPAAT, five unigenes encoding PLA2, three unigenes encoding CCT and two unigenes encoding CK were only weakly expressed in the SI-5 stage.

**Figure 8:**
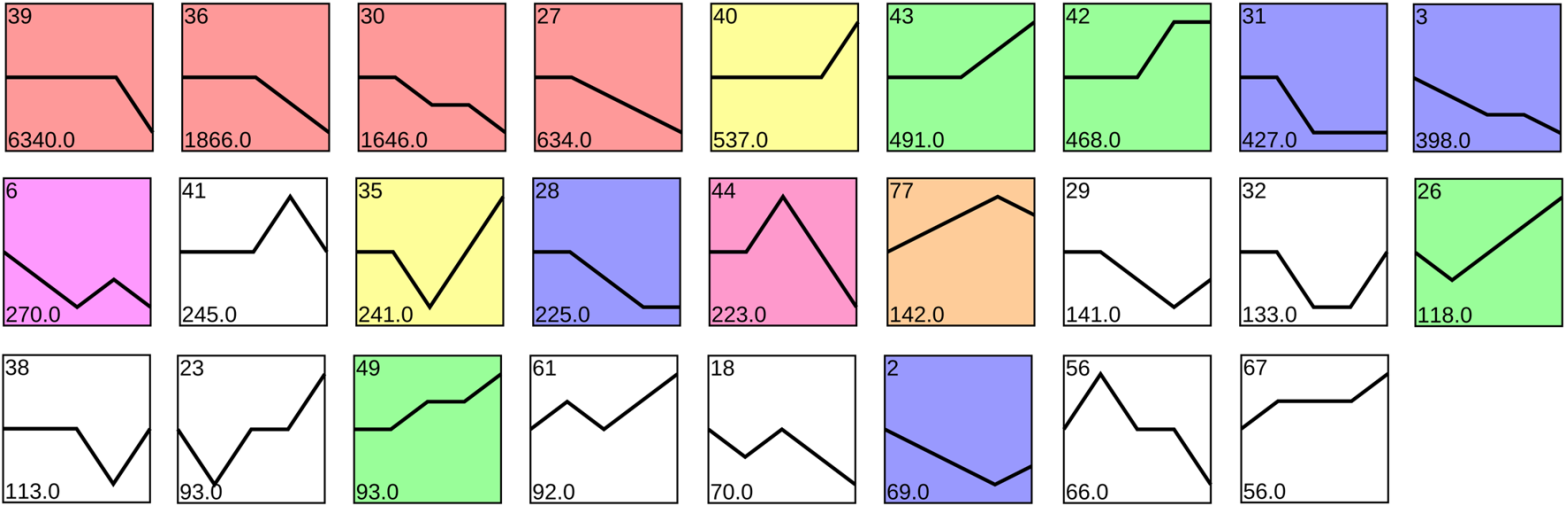
RNA-seq based transcriptome dynamics of 84 different expressed unigenes (68 of which are non-differentially expressed unigenes) related to ALA biosynthesis and TAG assembly through the 5 stages of sacha inchi seed development classified into 26 profiles. Note: blocks shaded with colours represent profiles with significant enrichment; blocks representing profiles with similar trends share the same color of shading; blocks without colour shading (i.e. white blocks) represent profiles without significant enrichment. The numbers in the bottom left corner of each block is the number of each profile.

Based on the analysis of ALA synthesis and TAG assembly, a total of 152 unigenes were identified as candidate genes for 30 enzymes (Supplementary Table S3). In order to examine trends in the expression of these 152 unigenes, the normalized expressed data of 20,446 DEGs were analysed with Short Time-series Expression Miner software (STEM) version 1.3.12. This resulted in the 20,446 DEGs being clustered into 80 groups or ‘profiles’, with the 84 unigenes of the DEGs (68 of the non-differentially expressed unigenes) which related to ALA and TAG biosynthesis being clustered into just 25 of these 80 profiles (Fig. 8 and Supplementary Table S4).

Of the 25 profile categories for RNA-seq based transcriptome dynamics of the 74 differentially expressed unigenes related to ALA biosynthesis and TAG assembly through the 5 stages of sacha inchi seed development, 18 of the patterns were significant while seven proved not significant (Fig. 8). From among these profiles, particularly noteworthy is that 22 unigenes and 11 unigenes were involved in profile 39 and profile 40 respectively, while profiles 30 and 43 contained six unigenes. Meanwhile, profile 39 that contained 22 unigenes related to ALA and TAG biosynthesis pathways appeared to be stable across stages SI-1 through to SI-4 and then showed down-regulation in the SI-5 stage. These latter 22 unigenes include 6 unigenes involved in ALA biosynthesis (Unigene0009173 encoding FAD6, Unigene0004302 encoding KAR, and Unigene0020640 encoding α-PDH and three unigenes encoding FATB) and 16 unigenes involved in TAG assembly (Unigene0043373 encoding CCT, Unigene0023308 encoding CPT, Unigene0025739 encoding PDAT, Unigene0000190 encoding PDCT, Unigene0016645 encoding PP, three unigenes encoding FAD2, four unigenes encoding GPAT and three unigenes encoding LPAAT). Unigenes included in profile 43 (six unigenes) were down-regulated during stages SI-1 through to SI-3, but then their expression increased during stage SI-4, and these included three unigenes (Unigene0019543 encoding BCCP, Unigene0039280 encoding FATB and Unigene0007906 encoding β-PDH) involved in ALA biosynthesis and three unigenes encoding OLE which are unique to oil bodies and are unusual proteins^31^. Profile 30 included six unigenes (Unigene0009027 encoding FAD2, Unigene0013583 encoding GPDH, two unigenes encoding CCT and two unigenes encoding GPAT) which were all candidate genes involved in TAG assembly, and their expression showed sustained down-regulation across SI-1 to SI-5 stages.

In contrast to the proceeding profiles, profile 40 contained 12 unigenes involved in ALA and TAG biosynthesis pathways that appeared to be stably expressed from stage SI-1 through to SI-4, but then increased sharply during the SI-5 stage. The expression pattern of these latter 12 unigenes was consistent with the dynamic changes of ALA content and total PUFAs content during the sacha inchi seed growth., with five of the 12 (Unigene0008403 encoding FATA, Unigene0014324 encoding DHLAT, Unigene0024371 encoding KASII, Unigene0012943 encoding SAD and Unigene0022151 encoding α-CT) being candidate genes in ALA biosynthesis and the other seven (Unigene0037808 encoding FAD2, Unigene0044238 encoding FAD3, Unigene0022660 encoding GPDH, Unigene0007846 encoding LPCAT, Unigene0010027 encoding Oleosin3, Unigene0016056 encoding PDAT and Unigene0000909 encoding PDCT) being candidates in TAG assembly.

### Quantitative Real-Time PCR validation

**Figure 9:**
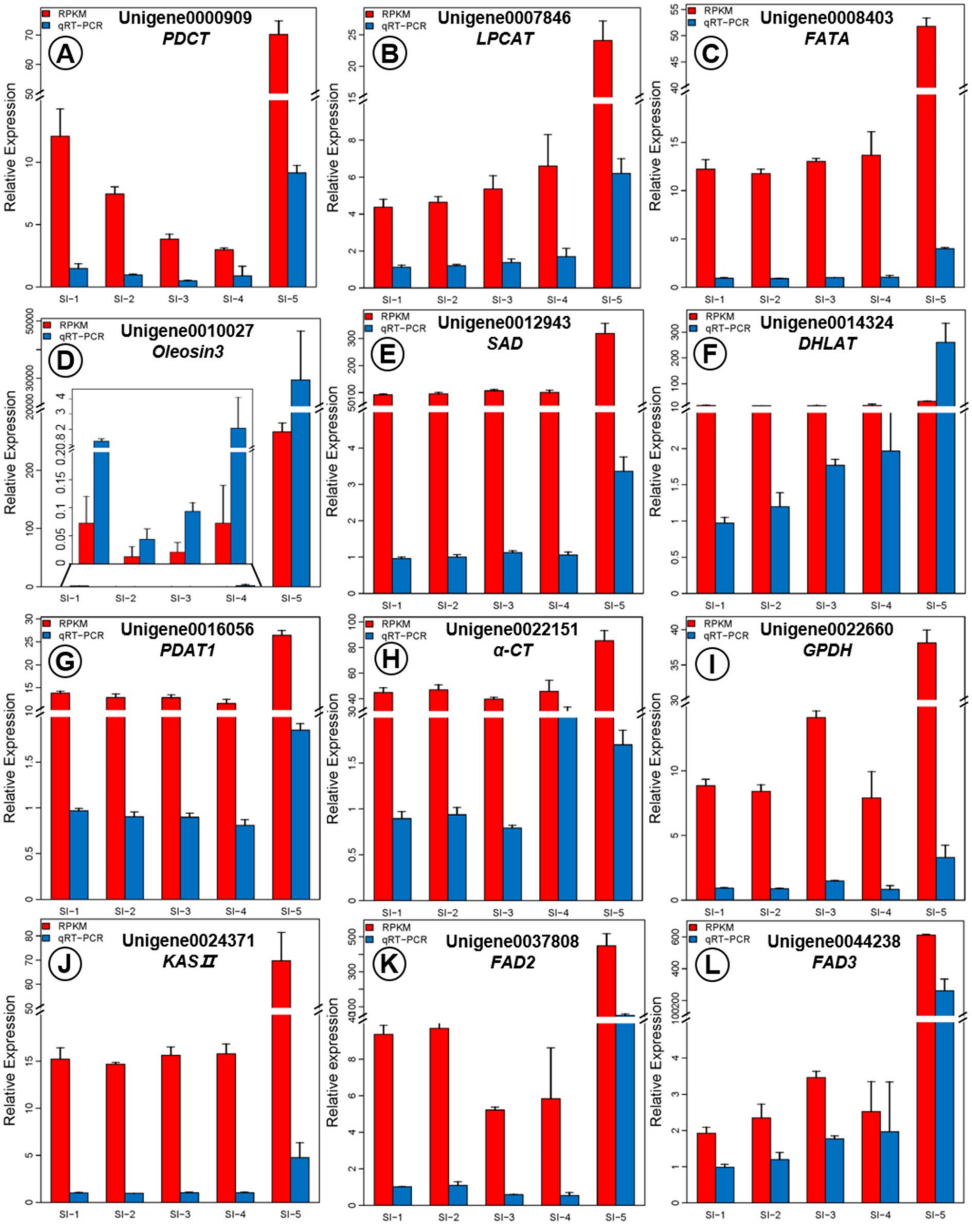
The expression levels of the 11 candidate unigenes related to ALA biosynthesis and PUFAs content as revealed by qRT-PCR and RNA-seq (**A**–**L**).

To further confirm the significance and accuracy of the 12 unigenes (Unigene 0000909, Unigene 0007846, Unigene0008403, Unigene0010027, Unigene0012943, Unigene0014324, Unigene0016056, Unigene0022151, Unigene0022660, Unigene0024371, Unigene0037808 and Unigene0044238) closely related to ALA biosynthesis and PUFA accumulation, the results from the transcriptome analysis (expression levels) were evaluated in each of the five stages of sacha inchi seed development using real-time quantitative reverse transcription PCR (qRT-PCR). This revealed that all 12 of these unigenes showed up-regulation commencing from the near-mature stage (SI-4) with a sharp increase of this up-regulated activity during the mature stage (SI-5) (Fig. 9). This observed pattern of activity is consistent with the RNA-seq data that also revealed increased expression of these unigenes. Both RNA-seq and qRT-PCR results showed that the activity of Unigene0010027 encoding Oleosin3, Unigene0037808 encoding FAD2 and Unigene0044238 encoding FAD3 were much higher at the SI-5 stage than in the other four stages, with the temporal profile of Unigene0044238 expression being in strong agreement with the ALA accumulation pattern during sacha inchi seed development suggesting its role in determining ALA content. Similarly, the expression pattern of the transcripts of FATA, SAD, DHLAT, PDAT1, α-CT and KASII in the results of RNA-seq and qRT-PCR were all stable prior to seed maturation and then increased dramatically at the mature stage (SI-5), consistent with the dynamic trend of total PUFAs accumulation across sacha inchi seed development.

**Figure 10:**
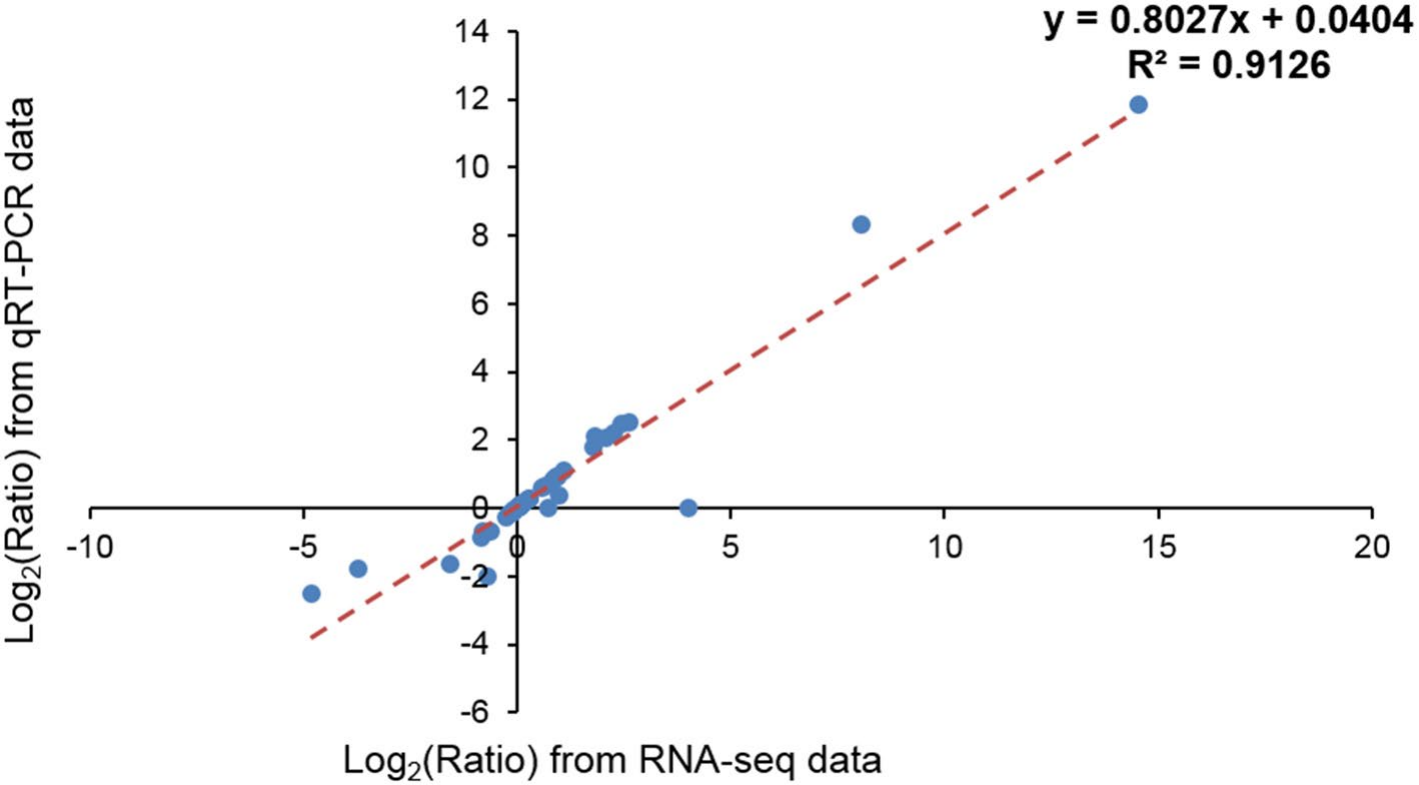
The correlation between qRT-PCR and the transcriptome data as shown by values of log_2_(relative expression ratios) in qRT-PCR (x-axis) plotted against the values of log_2_(RPKM ratios) in transcriptome data (y-axis) for the 12 candidate unigenes.

The above latter pattern indicates that RNA-seq results provided a reliable means for the identification and measurement of DEG expression during sacha inchi seed development, and it was clear that oleosin unigenes were expressed mainly during the later stages of seed development (i.e. SI-5—seed maturation stage). Meanwhile, a highly significant correlation (R^2^ = 0.9126) was found between the qRT-PCR results and the RNA-seq data for the 12 candidate unigenes (Fig. 10) which also suggests that the RNA-seq data are reliable and reflect the expression levels of the transcripts.

## Discussion

Seed development is a highly coordinated, genetically programmed and irreversible process that involves a series of physiological, biochemical, and organoleptic changes allowing for the development of a ripe seed^32^. In oil seed species, such development is accompanied by significant accumulation of fatty acids and carbohydrate degradation^33^. On account of sacha inchi’s ability to biosynthesize and accumulate considerable amounts of unsaturated fatty acids, especially ALA in its seeds^7^, the oil contained in its mature seeds has many potential applications in food, chemical and pharmaceutical industries.

Transcriptome sequencing has been applied to reveal key enzymatic unigenes regulating growth and development of many species, such as *Lentinula edodes*^34^, *Tussilago farfara*^35^, *Camellia oleifera*^36^, *Hippophae rhamnoides*^37^ and *Citrus sinensis*^38^. Such sequencing has also been used previously to analyse two developmental stages of sacha inchi seeds^7^. In this current study though, Illumina RNA-sequencing was used to analyse sacha inchi seeds at all five development stages and then to construct cDNA libraries from each of these five stages. Even so, the GC percentage content of each library in our study (average 43.6%) were consistent with Wang et al.’s^7^ earlier results (average 44.3%), though it must be noted that we collected samples with three biological duplications for RNA-seq analysis for each developmental stage and sequenced each sample just once as next generation sequencing data are highly repeatable.

In this current study the Q20 percentage, which represents the confidence level on the identification of bases during sequencing, reached more than 98% for each of the five cDNA libraries and the N50 value, which refers to the length of the smallest unigenes in the set that contains the fewest or largest unigenes whose combined length represents at least 50% of the assembly, was up to 2299 bp. Also, the E-value distribution of the annotated unigenes in Nr, Swiss-Prot, KEGG and KOG databases showed that there was strong homology (< 10^–20^) to available plant sequences (88.2%, 80.9%, 89.8% and 83.5%, respectively). And, the species distributions of the annotated unigenes showed that sequences from *R. communis* and *J. curcas* had the highest similarities; these species all belong to the same family (Euphorbiaceae) as sacha inchi. Taken together, these forgoing results indicate that the assembly and annotation of the transcriptome data completed by this study were reliable.

During seed development, in addition to fatty acid accumulation and carbohydrate degradation, there is a proliferation and expansion of embryo and endosperm cells involving nucleic acid synthesis associated with changes of enzyme activity as well as hormone regulation^31^. In this current study, the GO annotation of the 44,797 unigenes reported in a previous sacha inchi study^19^ illustrated that metabolic processes, catalytic activity and cellular processes were the top three categories by the number of unigenes. These three categories mainly relate to lipid metabolism, enzyme activity and cell proliferation and expansion respectively. Similarly, results of KEGG pathway-based analysis showed that carbohydrate metabolism and lipid metabolism mainly involved enrichment, carbohydrate degradation and fatty acid accumulation during development process of sacha inchi seed.

From the aspect of the potential gene functions, the biosynthesis of carbohydrate mainly occurred during early stages (SI-1 and SI-2) of seed development, the accumulation of proteins mainly occurred during middle and late stages (SI-3 and SI-4), and the accumulation of lipids mainly occurred during the maturation stage (SI-5), which concurs with results reported previously by both Liu et al.^9^ and Wang and Liu^39^.

Various other studies have shown that oil accumulation in developing seeds starts at the early stage and peaks at mid to late developmental stages and corresponds with a degree of dehydration of the maturing seeds^9,15^. In this current study, analyses of the expression of the core enzymes involved in ALA biosynthesis and TAG assembly showed that the transcripts of PDCT (Unigene0000909), LPCAT (Unigene0007846), FATA, Oleosin3 (Unigene0010027), SAD (Unigene0012943), DHLAT (Unigene0014324), PDAT (Unigene0016056), α-CT (Unigene0022151), GPDH (Unigene0022660), FAD2 (Unigene0037808), KAS II, and FAD3 were highly expressed during the course of *P. volubilis* seed development, especially at the mature stage (SI-5). Combined with the dynamic change of the main fatty acids across sacha inchi’s seed development, the expression trend of Unigene0044238 encoding FAD3 was highly consistent with the change in ALA content. The FAD3 in endoplasmic reticulum is the key enzyme, which introduces the third double bond, in the biosynthesis of 18:3 fatty acids^40^. Similarly, analyses of expression patterns of FAD3 in tree peony (*Paeonia ostii*) seeds, which also contain high levels of ALA (approximately 45% of the total content of unsaturated fatty acids), revealed that PsFAD3 has higher expression levels than SAD and FAD2 genes^41,42^. In sacha inchi, this current study found that Unigene0044238 encoding FAD3 was upregulated 279.43-fold in the mature stage (SI-5), indicating that this is likely one of the key unigenes in ALA biosynthesis in sacha inchi seed.

Additionally, the expression pattern of the seven unigenes encoding FAD2, FATA, SAD, DHLAT, PDAT1, α-CT and KASII were in line with the dynamic trend of total PUFAs content during seed development revealing their crucial roles in the formation of PUFAs in sacha inchi seed. For example, higher expression level of FATA in the seeds suggests the C18 fatty acids are one of the main products of plastidial FA synthesis, as FATA fulfills a fundamental role in the export of the C18 fatty acid moieties^43^. Previous studies showed that DGAT1 or PDAT1 were two genes confirmed essential for TAG biosynthesis, and their expression patterns vary among different species^44^. For example, during the biosynthesis of tung oil in seeds of the tung tree (*Venicia fordii*), the contents of LA and ALA are higher in young seed and gradually decrease as seed matures. Correspondingly, DGAT2 and PDAT are highly expressed in the earlier stages of seed development in this latter species^45^. In this current study, the transcripts of DGAT1and PDAT1 (Unigene0020593 and Unigene0016056, respectively) were upregulated in mature seed (SI-5) (Fig. 7B). Furthermore, the expression level of two OeFAD2 genes in olive (*Olea europaea*), which is a plant with higher OA and lower LA contents in its fruits, the content of ALA is almost negligible during the ripening (maturation) of its fruit^46^. Also in this current study, the expression of Unigene0037808 encoding FAD2 was upregulated 28.37-fold in the mature stage (SI-5) of sacha inchi seed development, suggesting FAD2 also has involvement in ALA biosynthesis in this species.

The enzyme KASII catalyzes the elongation of C16:0-ACP to C18:0-ACP and then SAD is an essential precursor that drives ALA synthesis by introducing the first double bond into C18:0-ACP to produce C18:1-ACP^47^. Consequently, the expression levels of KASII, SAD and FATA can affect the relative proportions of C18 fatty acids^48^. In *Symplocos paniculata*^21^, olive (*Olea europaea*)^46^ and avocado (*Persea americana*)^49^, higher expression levels of SAD have been correlated with the accumulation of C18:1. This current study also suggested that that SAD (Unigene0012943) has a central role in the accumulation of PUFAs in sacha inchi seeds. Additionally, transcription factors (TFs) or proteins can regulate the genes encoding core fatty acid synthesis. Numerous transcription factors are involved in a complex and hierarchical system integrating TAG production with other aspects of seed development^50^. Factor bZIP67 regulates ALA content by binding G-boxes in the FAD3 promoter of Arabidopsis^51^ and Liao et al.^52^ identified the transcription factor bZIP124, which might be a key factor to regulate the expression of *PvFAD3* gene.

The expression patterns of Unigene0006804, Unigene0035220 encoding bZIP43 were consistent with the change of ALA content during sacha inchi seed development in this current study, which might be closely related to the high content of ALA in seeds of this species. Two unigenes (Unigene0012060, Unigene0012061) encoding WRINKLED1 (WRI1) might directly control the transcriptional activation of fatty acid biosynthesis like in *Arabidopsis thaliana*^53^ and they were upregulated at the maturation stage (SI-5) consistent with the dynamic accumulation pattern of ALA. Also, 91 unigenes encoding MYB might directly repress the expression of the master regulator, WRI1, and that of the key genes in the ALA biosynthesis and TAG assembly pathway during sacha inchi seed development^54–56^.

Acylglycerols provide a key energy reserve in many organisms and are some of the common components of many seed oils^57–59^, with TAGs being the most common acylglycerol in such oils^21^. In mature seeds, the TAGs are stored in lipid bodies surrounded by oleosin proteins that occupy close to 60% of the cell volume of the cotyledons^60^. Usually, oil accumulation during seed development is associated with an increase in the number of lipid bodies^36^. Oleosin can be a reliable marker for such lipid bodies^27^ and it is usually expressed abundantly during seed development. For example, Huang^61^ and Jolivet et al.^62^ found that oleosin accounted for up to 79% of the total protein in *Arabidopsis* seeds. In this current study, expression profiles of unigenes associated with the lipid body protein oleosin3 (Unigene0010027) was analysed along with the dynamic changes in expression levels during the development of sacha inchi seeds. This revealed that the oleosin unigenes were highly expressed at the maturation stage of seed development in this species and that different oleosin unigenes had different expression profiles. We also used real-time PCR to verify the transcription profile of the twelve candidate genes related to ALA biosynthesis and TAG assembly revealed by RNA-seq data, with consistent expression trends indicating the RNA-seq data to be reliable.

## Conclusions

The present study has, for the first time, provided a dynamic view of the transcriptome during sacha inchi seed development. Transcriptome analysis identified many important enzymes with known roles in seed development, such as crucial enzymes involved in ALA biosynthesis and TAG assembly and an integrated pathway related to core lipid metabolism was reconstructed. In addition, the temporal expression levels of 12 unigenes encoding key enzymes were also validated using qRT-PCR. This identification of unigenes of specific functions provides a foundation for future studies on molecular mechanisms that regulate ALA biosynthesis and PUFAs accumulation in seeds of this species. Further research is needed to investigate these genes in order to understand the rate-limiting enzyme genes that are critical to achieving high ALA production through genetic breeding and/or other molecular genetic technologies.

## Methods

### Biological material

Sacha inchi plants grown from seeds sourced from the Peruvian Amazon Research Institute (IIAP) in San Martin, Peru were cultivated at South China Experimental Nursery, located in Zhanjiang city in the southwest of Guangdong province, China (longitude 111°38′E, latitude 21°30′N, and altitude 90 m above sea level). This site has a tropical monsoonal climate, with a mean annual mean temperature of 23.1 °C, absolute minimum temperature of 3.6 °C, absolute maximum temperature of 38.8 °C and mean annual rainfall of 1567 mm.

When the sacha inchi plants were approximately three years of age at the start of April 2017, observations commenced on flowering phenology of 15 plants and then on the fruit and seed development process, continuing through to late September 2017. When grown in Zhanjiang, this species flowers over a protracted period of 6 months or more, with fruit development and ripening occurring over similarly protracted periods. Development of all fruits/seeds was recorded by the number of days after pollination, with pollination nominally considered to have occurred on the day of anthesis, i.e. anthesis = 0 days after pollination (DAP). Within the 6-month period of observation, many mature seeds had been produced (at around 120 DAP), from flowers whose initiation had been observed the preceding April to May.

### Seed development stages and biological metrics

Five distinct stages in the development and maturation of sacha inchi seeds, based on changes in their relative contents of fatty acids have been identified by preceding studies^9,19^. These stages, which vary in length from 10 to 30 or more days are: the initial stage (SI-1, 0–10 DAP), second stage (SI-2, 20–40 DAP), third stage (SI-3, 50–70 DAP), fourth stage (SI-4, 80–110 DAP) and the fifth stage (SI-5 > 120 DAP). The periods or gaps between successive stages are transition periods.

For transcriptome sampling, seeds from all five development stages (as determined by DAP) were collected at around 11:00 a.m. on September 27, 2017. For each seed development stage, samples were collected from 3 ‘replicates’, with each ‘replicate’ comprising a different plant. The collected samples were flash frozen in liquid nitrogen and stored at − 80 °C until needed for analyses.

Major biological metrics for seeds from each of the five stages have been provided by a preceding study^19^ that used subsamples of the very same sacha inchi seeds collected for the study reported here. Details of oil contents of these seeds, especially the dynamic change of ALA content and the total PUFAs content, are provided in Supplementary Material Table S1.

### RNA preparation

The RNA from seeds from each of the five development stages were prepared separately using RNAprep Pure Plant Kit (TIANGEN, Beijing, China), then incubated with DNase I (RNase-free) (TaKaRa Bio, China) for 30 min at 37 °C to remove residual DNA. Quantities and qualities of total RNA were assessed using NanoDrop 2000 (Thermo Scientific, Wilmington, DE, USA) and RNase free agarose gel electrophoresis. The RNA integrity number (RIN) determined by the Agilent 2100 Bioanalyzer was greater than 9.0 for all samples.

### cDNA library construction and sequencing

After total RNA was extracted from each of the 15 lots of seeds (i.e. three lots—replicates—from each of the five stages of seed developmental), a GeneRead Pure mRNA Kit (QIAGEN) was used for polyA-oligo-dT–based purification of mRNA, in order to prepare it for sequencing. The enriched mRNA was then fragmented into short fragments using a fragmentation buffer and reverse transcripted into cDNA with random primers. Second-strand cDNA were synthesized by DNA polymerase I, RNase H, dNTP and buffer and these cDNA fragments were purified with Agencourt AMPure XP beads (1.8×), end repaired, poly(A) added, and ligated to Illumina sequencing adapters. The ligation products were size selected by agarose gel electrophoresis and PCR amplified. The cDNA library was sequenced using an Illumina HiSeq 4000 (Gene Denovo Biotechnology Co., Guangzhou, China).

### De novo assembly

Reads obtained from the sequencing machines included dirty reads containing adapters or low-quality bases that would affect subsequent assembly and analyses. In order to obtain high quality clean reads, the obtained reads were filtered by removing those containing adapters, those containing more than 10% of unknown nucleotides (N) and those of low quality containing more than 40% of low quality (Q-value ≤ 20) bases. In the absence of a reference genome, transcriptome de novo assembly was carried out using the short reads assembling program Trinity 2.8.6^63^. Transcriptome completeness was assessed using the Benchmarking Universal Single-Copy Orthologs software (BUSCO, version 3.0.2)^64^ that incorporates 1440 single-copy orthologous genes as the embryophyte dataset.

### Functional annotation

Basic annotation of unigenes included protein functional annotation, pathway annotation, Cluster of Orthologous Group (COG)/euKaryotic Ortholog Group (KOG) functional annotation and Gene Ontology (GO) annotation. To annotate and classify unigenes isolated from sacha inchi into these categories, we used the BLASTx program (https://www.ncbi.nlm.nih.gov/BLAST/, accessed on November 15th 2018) with an E-value threshold of 1e^−5^ to NCBI non-redundant protein (Nr) database (https://www.ncbi.nlm.nih.gov, accessed on December 6th 2018), the Swiss-Prot protein database (https://www.expasy.ch/sprot, accessed on December 10th 2018), the Kyoto Encyclopedia of Genes and Genomes (KEGG) database (https://www.genome.jp/kegg, accessed on December 13th 2018), and the COG/KOG protein database (https://www.ncbi.nlm.nih.gov/COG, accessed on December 17th 2018). Protein functional annotations were then obtained according to the best alignment results. GO annotation was performed with Blast2GO 5.2.5 (https://www.blast2go.com/)^65^ and GO functional classification was carried out using WEGO 2.0 (https://www.blast2go.com/)^66^ and GO functional classification was carried out using WEGO 2.0 (https://wego.genomics.org.cn/)^67^.

### Differential expression of genes (DEGs) analysis

The expression level of each unigene was calculated and normalized to RPKM (reads per kb per million reads)^67^. To identify DEGs across all five groups (i.e. five development stages), edgeR 3.10 software (https://www.r-project.org/)^68^ was used. Unigenes with a fold change (FC) ≥ 2 and a false discovery rate (FDR) < 0.05 were identified as significant DEGs.

To examine trends in the expression of the DEGs associated with oil accumulation (from the 10 pairwise comparisons among the five seed development stages), expressed data were normalized to means of zero following procedures described by Lu et al.^69^. DEGs were clustered by Short Time-series Expression Miner software (STEM) version 1.3.12^70^ and the clustered profiles with P-values ≤ 0.05 were considered as significantly expressed. The DEGs were then subjected to enrichment analysis of KEGG pathways; KEGG pathway-based analysis helps identify the biological pathways that are related to unigenes^71,72^.

### RT-qPCR validation of RNA-sequence data

RNA-sequence (RNA-seq) data was obtained using total RNA extracted from the sacha inchi seed samples using a RNAprep Pure Plant Kit (TIANGEN, Beijing, China) and reverse transcribed using a FastQuant RT Kit (with gDNase) (TIANGEN, Beijing, China). The primers used for this purpose are listed in Supplementary Table S5. The qRT-PCR amplifications were performed with a Thermo Scientific PikoReal 96 Real-Time PCR System using iQ SYBR Green Supermix (TaKaRa Bio, China) according to the manufacturer’s instructions. PCR product specificities were confirmed by melt-curve analysis. Triplicate samples of RT-qPCR for each unigene were prepared in a total volume of 20 μl that contained 4 μl cDNA template, 10 μl qPCR Master Mix and 0.4 μl in each of forward and reverse primers. The Actin gene (unigene 0026056) was used as an internal reference standard, and the relative expression ratio of target unigenes were calculated as described in the 2^−∆∆Ct^ method^73^. Raw reads sequenced using the RNA-Seq technique were filtered to remove adaptors, ambiguous nucleotides and low-quality sequences, to identify high quality (‘clean’) reads.

## Supplementary information

Supplementary Tables.

## Data Availability

The datasets used and/or analysed during the current study are available from the corresponding author on reasonable request.
